# Organizational Dehumanization, Perceived Stress, and Patient Depersonalization in French Nurses: A Cross‐Sectional Test of the Spillover Model

**DOI:** 10.1155/jonm/2207579

**Published:** 2026-06-22

**Authors:** Marie Charlotte Mollet, Oulmann Zerhouni, Corinne Isnard Bagnis, Lucia Romo

**Affiliations:** ^1^ Centre for Research on Psychological Functioning and Dysfunction (CRFDP), University of Rouen Normandy, Mont-Saint-Aignan, France; ^2^ EA 4430 Clinical, Psychoanalytic and Developmental Psychology (Clipsyd), Université Paris Nanterre, Nanterre, France, parisnanterre.fr; ^3^ Parisian Laboratory of Social Psychology (LAPPS/PS2C), Paris Nanterre University, Nanterre, France, parisnanterre.fr; ^4^ Department of Nephrology, AP-HP Sorbonne University, Paris, France; ^5^ Raymond-Poincaré University Hospital (AP-HP), Garches, France; ^6^ Inserm CESP U1018, Paris-Saclay University, Orsay, France, universite-paris-saclay.fr

**Keywords:** nurses, organizational dehumanization, patient dehumanization, perceived stress

## Abstract

**Objective:**

To examine the association between organizational dehumanization (OD) and nursing staff’s perceived stress and to test whether perceived stress statistically mediates the relationship between OD and patient depersonalization, as proposed by the spillover model.

**Design:**

Cross‐sectional online survey.

**Methodology:**

A total of 291 registered nurses in France completed an online questionnaire measuring OD, perceived stress, and depersonalization. Mediation analysis with bias‐corrected bootstrapping was used to examine the hypothesized pathway linking OD to patient depersonalization through perceived stress.

**Results:**

OD is significantly associated with perceived stress. Perceived stress functioned as a partial and statistically significant mediator in the relationship between OD and depersonalization. A significant direct effect of OD on depersonalization persisted, suggesting that stress only partially explains the relationship.

**Conclusions:**

The findings provide cross‐sectional support for the spillover hypothesis: OD is associated with psychological strain which in turn is associated with depersonalized attitudes toward patients. These findings suggest that OD may be part of a stressful work climate in which depersonalization is more likely to be observed, although causal interpretations cannot be drawn.

**Impact:**

The results highlight OD as a potential psychosocial risk factor for nurses and as a contextual correlate of depersonalization in patient care. Policies should be informed by further longitudinal and intervention studies before drawing firm conclusions about mechanisms or causality. Also, interventions should prioritize reducing the perceived stress of staff and restoring the ethical quality of the work environment over merely managing individual stress.

**Implication for Nursing Management:**

These findings suggest that nursing managers should address OD as a contextual risk factor by reducing dehumanizing organizational practices and strengthening ethical, resource‐preserving work conditions, rather than relying solely on individual stress‐management strategies.

## 1. Introduction

For the past 2 decades, research has extensively highlighted the profound changes in the French hospital sector stemming from a succession of reforms initiated since the early 20th century [1]. The 1980s reforms constitute a major turning point, characterized by the increase of economic reasoning in healthcare management: they introduced a systematic cost/benefit analysis [2] and the adoption of accounting indicators. This imperative of cost control has led to constant pressure to make the hospital more efficient and less expensive [3], driving the application of a quantitative mode of thinking to care services [4] and the standardization of medical practices [5].

Today in France, the hospital operates under a managerial logic historically typical of commercial enterprises. This economic reasoning has transformed care by subjecting it to an industrial perspective [6]. The result is a rationalization of activity—through certifications, task standardization, and work automation—which imposes a more “mechanistic” functioning on all professional bodies.

Although this dynamic is accompanied by initiatives aimed at improving the quality of care [7], where quality now encompasses risk prevention, continuity of care, and “organizational” quality [8], it creates a fundamental tension. These quality approaches are, in fact, often perceived by health professionals as incompatible with the very principle of care [9].

Consequently, this process leads to demotivating and demoralizing health professionals and consequently leads to a dehumanization of both patients and those who work for the organization [10].

Nursing staff, in particular, acutely feel this economic and managerial focus as a dehumanizing approach from their institution. Indeed, the routinization of tasks, fragmentation of labor, and the perceived emotional distance of nurse managers from staff nurses constitute unfavorable occupational elements that may collectively contribute to the development of organizational dehumanization (OD) [11].

## 2. Conceptual Framework

### 2.1. From Dehumanization to OD

Dehumanization is a psychological phenomenon whereby individuals are perceived as fundamentally lesser or different, resulting in the denial of their human characteristics [12, 13]. Rooted in the study of extreme violence such as war and genocide, the concept historically sought to understand how perpetrators engage in egregious acts [14]. Research suggests that this process, described by Bandura [15], enables otherwise reprehensible conduct by devaluating or blaming the victim.

Haslam [12] proposes a dual‐mode model of dehumanization, based on the denial of two distinct sets of human qualities. Mechanistic dehumanization involves the denial of “Human Nature” (HN), resulting in the perception of the individual as an object, a robot, or a machine, lacking universal traits such as warmth, curiosity, or emotional responsiveness, and animalistic dehumanization stems from the denial of “Unique Humaness” (UH), perceiving the individual as immature or an animal, stripped of attributes like culture, rationality, or morality. Research indicates that may, in certain contexts, be adopted as an emotional coping strategy by caregivers to prevent burnout [16, 17].

Dehumanization is now recognized as an everyday phenomenon, notably within the workplace [12]. As an extension of this perspective into the corporate environment, the concept of OD focuses on the employee’s perception of the treatment received from their organization [18]. OD is subsequently defined as the subjective feeling of the employee being objectified by the organization, stripped of their subjectivity, and reduced to a mere tool serving the company’s objectives.

The repercussions of OD are multifaceted and detrimental to both the individual and the organization:

• Impaired well‐being and health: Employees perceiving OD reports significantly lower levels of well‐being and health [19], manifesting as increased burnout, anxiety, and somatic complaints [20, 21], low job satisfaction, more emotional exhaustion, reduced empathy, low organizational affective commitment, and more intentions to quit the organization [22, 23].
• Self‐objectification: OD induces a phenomenon of self‐objectification, where the employee internalizes the organization’s utilitarian perception and views themselves as an instrument rather than a complete human being [20].
• Spillover effect and depersonalization: Studies have highlighted that the dehumanizing treatment experienced by an employee can be transferred to the recipients of their services. For instance, OD experienced by prison officers may lead to the depersonalization of incarcerated persons, who are then perceived as objects or numbers, mirroring the initial organizational treatment [24].

The detrimental link between employee mistreatment and work stress is well‐established, impacting individuals’ psychological systems, negatively altering their perspectives, and reducing their available resources [25].

OD should not be conflated with other negative states such as burnout or depersonalization. Whereas OD refers to employees’ perception of being treated as mere instruments by the organization, burnout describes a syndrome of emotional exhaustion, cynicism, and reduced professional efficacy, and depersonalization specifically targets the relational stance toward care recipients. Self‐objectification, in turn, denotes the internalization of the organization’s instrumental view and represents a psychological process through which OD may foster burnout and depersonalization, rather than a synonym of OD itself.

### 2.2. OD, Occupational Stress, and the Caregiving Context

Within the high‐demand environment of healthcare, this relationship is particularly salient. OD appears as an important intermediary in the work–stress dynamic: existing studies have shown that OD is associated with higher levels of job stress and, in turn, with more frequent deviant behaviors among nurses. Importantly, the effect of OD on stress is moderated by occupational self‐efficacy, suggesting that a strong belief in one’s professional ability can weaken the psychological impact of OD [26].

### 2.3. Structural Demands, Stress, and Psychological Distress

These critical stressors are rooted in the structural demands of the nursing role. High levels of stress among nurses are frequently fueled by a combination of occupational factors, such as excessive working hours, heavy workloads, and chronic exposure to illness and death. These demanding clinical settings are characterized by psychosocial risks, which the World Health Organization (WHO) [27] and Leka et al. [28] define as features of work design, organization, and social context with the potential to cause psychological or physical harm. Specific examples of these risks, including excessive workload, limited scope for decision‐making, and poor team relations, significantly contribute to the elevated stress levels experienced by nursing staff. Consistent with this, the WHO [29] defines work‐related stress as the adverse reaction that occurs when there is a perceived mismatch between job demands and an individual’s skills and knowledge, particularly when workplace pressures exceed their capacity to cope.

Empirical evidence from the French context strongly supports the predictive role of demands in psychological distress. A recent cross‐sectional study of registered nurses in France identified two distinct latent profiles based on occupational demands, psychological symptoms, and psychological flexibility [30].

Crucially, one profile, marked by a significantly higher workload, greater emotional demands, and increased psychological distress and burnout, clearly demonstrates that high work intensity and emotional demands are robust predictors of negative psychological symptoms in nursing staff. Conversely, the second profile demonstrated lower demands and symptoms, suggesting a direct dose‐response relationship between occupational demands and distress. These person‐centered findings highlight that the combination of high demands (which align with the stressors leading to OD) and resulting psychological distress is a central challenge for this workforce, necessitating targeted organizational adjustments.

To integrate these elements, we draw on the Conservation of Resources (COR) theory [31]. The COR theory conceptualizes stress as a reaction to actual or threatened loss of valued resources or to insufficient resource gain following investment. In this perspective, OD can be understood as a structural condition that signals to nurses that their resources (e.g., dignity, recognition, autonomy, and emotional energy) are expendable, thereby triggering a loss spiral that fuels perceived stress and, over time, burnout and depersonalization. Our model therefore assumes that OD operates as a contextual resource‐depleting factor, while perceived stress represents the proximal expression of ongoing resource loss, which may ultimately spill over into depersonalized attitudes toward patients.

### 2.4. The Spillover Effect: From Employee Stress to Patient Depersonalization

Depersonalization, a key subcomponent of burnout [32], is conceptualized as a mechanism of moral disengagement. Within challenging contexts, such as hospitals, this involves “regarding the persons… with a level of detachment and indifference that is ordinarily reserved for inanimate objects” ([33], p. 308). As a moral disengagement strategy, depersonalization is often considered a milder form of dehumanization [34]. In essence, this positions depersonalization (the denial of subjectivity toward patients) as the individual, patient‐directed correlate of OD (the denial of subjectivity experienced by nurses from their institution).

In addition, findings confirm that the perception of OD experienced by prison officers predicts the development of depersonalized relationships with incarcerated persons. This negative spillover effect occurs because the relationship between OD and depersonalization is mediated by the officers’ level of job satisfaction [24].

In the healthcare context, this spillover is particularly concerning, as it may compromise the quality and ethics of care. High levels of work‐related stress associated with organizational demands appear to foster emotional coping strategies among nurses, which can be linked to increased depersonalization and burnout.

Empirical evidence confirms that according a lower human status to patients works as a defensive coping mechanism, protecting nurses from stress. A study by Trifiletti et al. [35] found that stress reduction is achieved through both the denial of UH traits (e.g., rationality and morality) and the attribution of Non‐UH (NUH) traits (e.g., instinct and impulsiveness). Specifically, nurses reported lower stress when patients were perceived in terms of instinct and drive (NUH) rather than rationality and morality (UH). This short‐term psychological functionality makes the patients’ suffering more bearable, but the denial of full humanity can impair communication and lead to negative patient reactions, which may paradoxically increase nurse stress over time.

Drawing on the work of Hoogendoorn and Delgado [36], this defensive dehumanization strategy is not solely an individual failure but is firmly rooted in structural and organizational failures (e.g., unsustainable workload and lack of autonomy). While the mechanism allows staff to achieve psychological distancing and maintain lower stress levels for continued short‐term performance, the authors strongly advocate for future research to move beyond individual psychology and integrate the organizational level of analysis to address the systemic causes that compel medical staff to dehumanize patients in the first place, thereby improving both provider well‐being and patient care quality.

Crucially, studies focusing on the psychological impact on nursing staff conclude that the root of patient dehumanization originates in the nurses’ self‐dehumanization [37]. When professionals internalize the organization’s mechanistic view of their role, it results in an inability to feel emotions, impersonality of care, and sterility of assistance. These behaviors, which are adopted as psychological protection mechanisms against stress, translate directly into the denial of the patient’s subjectivity. Therefore, the manner in which nurses perceive themselves—and are perceived by their institution—directly influences their capacity to engage ethically and empathetically, leading to the depersonalization of the patient. This underscores that OD is not merely a personnel issue but a systemic failure with direct, detrimental consequences for the recipients of care.

Indeed, previous studies have examined the impact of OD on employees’ outcomes in general, such as work engagement, perceived stress, and well‐being, but mostly outside of nursing populations (e.g., [19, 21]). More recently, Abou Zeid et al. [38] documented a link between OD, work stress, and engagement among nurses, thereby extending this literature to healthcare settings. Other research has shown that nurses’ work stress and emotional strain are negatively associated with care quality and are linked to more detached or depersonalized attitudes toward patients (e.g., [20, 39–41]). Taken together, this work suggests that OD may ultimately affect the nurse–patient relationship through increased psychological strain. However, to our knowledge, no study has explicitly tested this spillover mechanism in a nursing context by modeling how OD transmits its effects to patient depersonalization via perceived stress.

### 2.5. Aims

The preceding conceptual framework establishes that the modernization of the French hospital system has created a high‐stress organizational environment characterized by OD. This system has been profoundly reshaped by New Public Management‐inspired reforms (activity‐based funding, performance indicators, and cost‐containment policies), which intensify work and promote a more instrumental view of both staff and patients. In this context, nurses are particularly likely to experience OD, making this healthcare setting a theoretically relevant case for examining how OD relates to stress and patient depersonalization.

While the literature documents associations between OD and stress‐related outcomes among nursing staff, the proposed mediational pathway from OD to patient depersonalization through perceived stress has not yet been empirically tested in the French context.

From a COR perspective, OD signals to nurses that their core resources—such as dignity, recognition, autonomy, and emotional energy—are threatened or expendable. Such structural signals are likely to trigger a sense of resource loss and to increase perceived stress as nurses struggle to cope with escalating demands in a context where they feel treated as mere instruments. In turn, sustained stress and resource depletion can foster burnout‐related responses, including depersonalization, which allows nurses to distance themselves emotionally from patients in order to conserve what remains of their psychological resources.

On this basis, the present research is structured around two primary objectives:

• To examine the association between OD and nursing staff’s perceived stress.
• To test the hypothesized mediating role of perceived stress in the relationship between OD and patient depersonalization, consistent with the theoretical model of the spillover effect (OD ⟶ stress ⟶ patient depersonalization)

## 3. Methods

### 3.1. Design

For this study, we employed a cross‐sectional survey methodology. Given the cross‐sectional design of this study, the mediation model should be interpreted as reflecting theoretically coherent associations rather than demonstrating temporal or causal processes. Although our hypothesised pathway from OD to perceived stress and then to patient depersonalization is grounded in prior theory, our data do not allow us to establish the temporal ordering of these variables or to rule out reciprocal influences. Data were collected via an anonymous online questionnaire administered between June and August 2024.

### 3.2. Report Transparency

This manuscript was prepared in accordance with the STROBE Statement guidelines for observational studies (Appendix 1).

### 3.3. Participants

The participants comprised 291 registered nurses practicing in France, recruited via the social media platforms Facebook, Instagram, and LinkedIn. A snowball sampling strategy was implemented whereby initial nurse contacts were invited to share the survey with their professional peers. This approach was chosen because direct access to nurses through hospital administrations is often restricted and because our objective was to reach nurses working across a wide range of hospitals, units, and employment statuses rather than to obtain a statistically representative sample of all French nurses. We acknowledge that this nonprobability sampling strategy may introduce self‐selection bias and limit the generalizability of our findings. However, it is commonly used in research on hard‐to‐reach professional groups and in studies addressing sensitive topics such as organizational mistreatment, where anonymous online participation can facilitate disclosure. As a result, the study population represents a broad geographical and institutional spectrum, including nurses from various hospitals throughout France. Participation was restricted to actively employed registered nurses. Of the 499 individuals who accessed the online questionnaire, 291 (58.3%) provided sufficiently complete responses, which were defined as completing all core survey modules, and they were retained for analysis. Fewer than 5% of data points were missing within this analytic sample of 291 participants. Data collection occurred from June to August 2024.

Questionnaires were excluded if an entire scale was left blank. However, cases with a single missing item within an otherwise completed scale were retained for analysis. Eighty‐eight participants (17.6%) discontinued the questionnaire during the first scale. An additional 15 participants (2%) completed the survey but did not provide their socio‐demographic data. The remaining participants who did not complete the survey (22%) discontinued at various points in the middle of the questionnaire. The final dataset for the 291 participants contained less than 5% missing data, which was handled using mean imputation.

### 3.4. Ethical Considerations

All study procedures followed the ethical principles of the 1964 Declaration of Helsinki and its later amendments. As the study was noninterventional and posed minimal risk to participants, it was classified under the French ‘Loi Jardé’ framework (RIPH3, Code de la santé publique, Articles L1121‐1 to L1128‐12). In accordance with these national regulations, this type of research does not require formal approval by a research ethics committee.

#### 3.4.1. Data Protection and Consent

The protocol was reviewed by the Data Protection Officer of the University of Rouen Normandie for compliance with GDPR requirements. No separate ethics committee approval or registration number was required under the applicable French regulatory framework. Participants provided informed consent prior to their involvement. This work was unfunded; the authors were solely responsible for all research stages, from design and data collection to analysis and publication decisions.

#### 3.4.2. Data Availability Statement

The datasets generated and analyzed during the current study are not publicly available due to data protection and GDPR constraints but are available from the corresponding author on reasonable request and subject to appropriate data‐sharing agreements.

### 3.5. Procedure and Data Collection

Recruitment was conducted in France through social media platforms (Facebook, Instagram, and LinkedIn) using a Qualtrics‐based survey. Eligible participants were adult registered nurses (≥ 18 years old) working in various healthcare environments, including hospitals, nursing homes, and private sectors. A final sample of 291 participants was obtained from 499 initial entries. The dataset showed high integrity, with missing data rates consistently below 5% for all study variables.

### 3.6. Data Analysis

The collected data were analyzed using the statistical software Jamovi (Version 2.5.5) (The Jamovi Project 2025a), which is based on R (Version 4.5). Prior to the main analyses, descriptive statistics (means, standard deviations, ranges, and frequencies as appropriate) were computed for all study variables to examine their distributions and identify potential outliers (Table 1). The reliability of the multi‐item scales (organizational dehumanization, perceived stress, and depersonalization) was assessed using Cronbach’s alpha (*α*). Additionally, Harman’s single‐factor test was conducted to evaluate the potential for common method variance.

**TABLE 1:** Descriptive analysis.

Variable	%	M	SD	Min	Max
Demographics					
Female	90.7				
Male	8.2				
Age (Years)		33.21	8.42	19.00	57.00
Seniority (Years)		8.36	7.49	1.00	35.00
Study variables					
Organizational dehumanization (OD)		3.80	0.80	1.55	5.00
Perceived stress (PSS‐10)		2.19	0.53	0.60	3.40
Depersonalization		2.25	1.37	0.00	6.00

Bivariate relationships were first explored using Pearson correlation coefficients among all continuous variables. Before proceeding with inferential testing, the assumptions of linear regression were verified. The independence of residuals was confirmed using the Durbin–Watson test, and multicollinearity was assessed using the Variance Inflation Factor (VIF). The assumptions of normality of residuals (Shapiro–Wilk test and Q‐Q plots) and homoscedasticity (scatter plot of standardized residuals versus fitted values) were also evaluated.

The proposed mediation model OD > perceived stress > depersonalization was performed using the General Linear Model (GLM) Mediation module within jamovi (Version 2.5.5), following the established methodology for testing indirect effects. Given the large sample size and observed violations of the assumptions of normality and homoscedasticity in preliminary testing, the significance of the indirect effect was robustly estimated using the bias‐corrected bootstrapping (BCa) procedure with 5000 resamples. The indirect effect was deemed significant if the 95% confidence interval did not contain zero.

### 3.7. Materials

#### 3.7.1. OD

OD was measured using the scale developed by Caesens et al. [21]. The French version consists of 11 items. These affirmations capture the perception of being treated impersonally by one’s organization, with examples including, “My organization considers me as a tool dedicated to its own success” and “My organization treats me as if I were an object.”

Participants indicated their level of agreement with each statement using a 7‐point Likert‐type scale, ranging from 1 (*strongly disagree*) to 7 (*strongly agree*). In the present sample, the scale showed high internal consistency with a Cronbach’s alpha of 0.90.

#### 3.7.2. The Perceived Stress Scale (PSS)

Perceived stress levels were measured using the 10‐item PSS (PSS‐10) [42]. This self‐report instrument utilizes a Likert scale ranging from ‘never’ to ‘very often’ to assess the frequency of stressful experiences over the past month. The scale includes six reverse‐scored items.

The PSS‐10 has consistently demonstrated strong psychometric properties, including a meta‐analytic mean internal consistency coefficient of 0.83 and mean test–retest reliability of 0.72 over a 1‐month interval [43]. The French adaptation [44] confirmed the original two‐factor structure and supported its validity: it correlated positively with the General Health Questionnaire (*r* = 0.58) (convergent validity) and negatively with the Life Orientation Test (*r* = −0.45) (divergent validity).

In the present sample, the scale showed high internal consistency, with a Cronbach’s alpha of 0.80, indicating that it reliably captured perceived stress in our participant group.

#### 3.7.3. Maslach Burnout Inventory (MBI)

The depersonalization dimension of burnout was assessed using the relevant subscale from the 22‐item MBI [45].

The depersonalization subscale was employed, which reflects a detached, negative, and impersonal reaction toward the recipients of one’s care or service. This dimension is scored on a six‐point frequency scale ranging from 0 (*never*) to 6 (*daily*).

The subscale has demonstrated robust psychometric properties in hospital‐based nursing samples, with a meta‐analytic mean Cronbach’s alpha of 0.79 [46]. In the present sample, the internal consistency for the depersonalization subscale was 0.70.

## 4. Results

### 4.1. Participants

Participant ages spanned from 19 to 57 years, with an average age of 33. The sample was predominantly female, with 264 women (90.7%), 24 men (8.2%), and 2 who did not disclose their gender (0.7%). Our sample’s gender composition reflects the broader professional landscape, consistent with the 2017 statistical data from the French Ministry of Health (DREES) and Santé publique France. Years of experience varied from 1 to 35 years among the participants. Table 1 provides a summary of the participants’ characteristics.

Given that all variables were collected using self‐report measures at a single point in time, we conducted Harman’s single‐factor test to assess the potential threat of common method bias. All items corresponding to the three main constructs (i.e., OD, perceived stress, and depersonalization) were entered into an unrotated principal component analysis. The results showed that the first factor accounted for only 25.6% of the total variance. As this value is well below the 50% threshold commonly suggested in the literature [47], common method bias does not appear to be a significant concern in the current study.

Building on Harman’s test, we conducted an unmeasured latent method factor (ULMC) analysis following Williams et al. [48]. We first fitted a baseline three‐factor confirmatory factor analysis (OD, PSS, and depersonalization), which fitted the data substantially better than a one‐factor solution (Δχ^2^(3) = 626.0, *p* < 0.001), supporting the empirical distinctiveness of the three constructs. We then estimated a Method‐C ULMC model, in which a single method factor with loadings constrained to be equal across all 26 items was added orthogonally to the substantive factors. The method factor accounted for an average of 17.9% of item variance. Critically, the directional pattern of substantive associations was preserved when method variance was controlled: the OD–PSS correlation was essentially unchanged (0.323 vs 0.318), the OD–depersonalization correlation showed modest attenuation (0.251 vs 0.226), and the PSS–depersonalization correlation declined from 0.205 to 0.155. Together with the poor fit of the one‐factor model (CFI = 0.57, RMSEA = 0.12), these results indicate that common method variance is unlikely to be the primary driver of the observed associations, although it does account for a nontrivial share of item variance in PSS–depersonalization specifically. We report this analysis as a more sensitive complement to Harman’s test, while acknowledging that the absence of a designed marker variable in our protocol prevents a fully rigorous correction of method bias [49].

### 4.2. Correlation Matrix

The correlation matrix presented in Table 2 provides insight into the linear relationships between the demographic variables (gender, age, and seniority) and the key psychological constructs (OD, PSS‐10, and depersonalization).

**TABLE 2:** Correlation matrix.

	Gender	Age	Seniority	OD	PSS 10	Depersonalization
Gender	—					
Age	0.059	—				
Seniority	0.078	0.753^∗∗∗^	—			
OD	0.189^∗∗^	0.023	0.034	—		
PSS 10	0.103	−0.192^∗∗^	−0.132^∗^	0.265^∗∗∗^	—	
Depersonalization	−0.190^∗∗^	−0.250^∗∗∗^	−0.188^∗∗^	0.239^∗∗∗^	0.190^∗∗^	—

^∗^ *p* < 0.05, ^∗∗^ *p* < 0.01, ^∗∗∗^ *p* < 0.001.

#### 4.2.1. Relationships Between Study Variables

• OD and perceived stress (PSS‐10): A significant positive correlation was observed between OD and PSS‐10 (*r* = 0.265, *p* < 0.001). This indicates that higher perceptions of OD are strongly associated with higher levels of perceived stress.
• OD and depersonalization: A highly significant positive relationship was found between OD and depersonalization (*r* = 0.239, *p* < 0.001). This result suggests that employees who feel treated impersonally by their organization (higher OD) are more likely to exhibit cynical and detached attitudes toward the recipients of their care (higher depersonalization).
• Perceived stress (PSS‐10) and depersonalization: A significant positive correlation exists between PSS‐10 and depersonalization (*r* = 0.190, *p* < 0.01). This finding suggests that higher perceived stress levels are moderately associated with increased levels of depersonalization.

### 4.3. Regression

A simple linear regression was performed to explore the extent to which OD is associated with perceived stress (PSS‐10) (Table 3).

**TABLE 3:** Linear regression.

**Model fit**				

**Model**	***R***		**R** ^2^	

1	0.265		0.0703	

**Model coefficient-PSS 10**				

**Predictor**	**Estimation**	**Standard error**	***t***	**p**

Intercept	1.524	0.1465	10.40	< 0.001
OD	0.176	0.0377	4.67	< 0.001

The model successfully accounted for a portion of the variability in perceived stress, demonstrating a correlation of 0.265 and an explanatory power of 0.070. Consequently, 7.0% of the variance observed in perceived stress is attributable to OD.

Examining the predictor coefficients confirmed that OD is significantly associated with perceived stress (*t* = 4.67, *p* < 0.001). The positive unstandardized coefficient (*β* = 0.176) reveals a direct relationship where heightened perceptions of OD are associated with higher levels of perceived stress.

### 4.4. Mediation

A mediation analysis was conducted to examine whether the association between OD and depersonalization is statistically mediated by perceived stress (Table 4).

**TABLE 4:** Mediation.

Indirect and total effects								
Type	Effect	Estimate	SE	95% C.I. (a)		Β	z	*p*
				Lower	Upper			
Indirect	OD ⟹ PSS 10 ⟹ Depersonalization	0.0620	0.0297	0.00764	0.137	0.0362	2.09	0.037

Component	OD ⟹ PSS 10	0.1764	0.0376	0.09425	0.257	0.2651	4.69	< 0.001
	PSS 10 ⟹ Depersonalization	0.3515	0.1507	0.00364	0.674	0.1364	2.33	0.020

Direct	OD ⟹ Depersonalization	0.3487	0.1003	0.13452	0.558	0.2033	3.48	< 0.001

Total	OD ⟹ Depersonalization	0.4107	0.0978	0.19864	0.612	0.2394	4.20	< 0.001

*Note:* Confidence intervals computed with method: Bias‐corrected bootstrap. Betas are completely standardized effect sizes.

The total effect of OD on depersonalization was statistically significant and positive (*β* = 0.2394, *p* < 0.001), indicating that higher perceptions of being dehumanized by the organization are associated with greater depersonalization toward patients.

In the final regression model predicting patient depersonalization from both OD and perceived stress, the overall amount of explained variance was modest. Together, these predictors accounted for approximately 7.5% of the variance in depersonalization (*R* ^2^ = 0.07), indicating that OD and perceived stress represent only a small portion of the multiple factors likely to shape nurses’ depersonalized attitudes toward patients.

#### 4.4.1. The Mediation Pathway

The analysis of the indirect path through PSS‐10 demonstrated a significant mediation effect.

Firstly, the relationship between the independent variable and the mediator was established, confirming that OD is significantly associated with perceived stress (*β* = 0.2651, *p* < 0.001). Secondly, the mediator is significantly associated with the dependent variable, as perceived stress is significantly associated wtih depersonalization when controlling for OD (*β* = 0.1364, *p* = 0.020).

Crucially, the indirect effect of OD on depersonalization through perceived stress was statistically significant, as the BCa confidence interval did not include zero (*B* = 0.0620, 95% [0.00764, 0.137], *p* = 0.037). This result suggests that perceived stress is a significant statistical pathway through which OD is associated with higher levels of depersonalization.

#### 4.4.2. Direct Effect

While the indirect effect was significant, the direct effect of OD on depersonalization remained significant when controlling for perceived stress (*β* = 0.2033, *p* < 0.001).

The retention of a significant direct effect suggests a pattern of partial mediation: perceived stress only partially accounts for the association between OD and depersonalization, and OD remains directly associated with depersonalization beyond this indirect pathway.

## 5. Discussion

This research was primarily designed to rigorously examine the hypothesized psychological pathway linking systemic organizational failings to adverse staff and patient‐directed outcomes within the highly demanding French hospital environment. Our first objective was to empirically quantify the extent to which nurses’ perceptions of OD are associated with their perceived stress (PSS‐10) levels. Subsequently, the core goal was to test the spillover effect, specifically by investigating whether perceived stress functions as a key statistical pathway through which experiences of OD are associated with depersonalizing attitudes toward patients. The ultimate aspiration of this clarification of the theoretical model was to yield targeted organizational insights for mitigating the systemic roots of dehumanization. Consistent with our theoretical framework, the findings confirm that OD is significantly associated with perceived stress, and most critically, that perceived stress acts as a partial but statistically significant mediator in the relationship between OD and depersonalization.

### 5.1. Stress and Dehumanization

Current research indicates that OD has detrimental effects on employee health, and our study contributes new knowledge regarding its direct link with perceived employee stress. Specifically, our regression analysis reveals a relatively weak yet significant incidence of OD on the perceived stress experienced by our population of nursing staff.

This finding resonates with several recent studies demonstrating that OD is positively associated with occupational stress among nursing staff [38], as well as among employees in the tertiary sector [50].

Collectively, these results underscore, firstly, the critical importance of considering this phenomenon in the understanding of occupational distress among healthcare providers, and secondly, that it represents a mobilizable lever for the improvement of their working conditions.

### 5.2. The Spillover Effect

The observed defensive dehumanization is, as highlighted by Hoogendoorn and Delgado [36], deeply rooted in structural, rather than merely individual, failures, thereby emphasizing the crucial need to investigate the organizational context. Several studies have previously addressed this organizational dimension. For example, research has shown that work stress is negatively associated with the provision of care, particularly in connection with conflicts involving supervisors, families, patients, or colleagues [39]. This link between work stress and quality of care is corroborated across various studies [40, 41]. These findings serve as strong indicators that caring behaviors, and by extension, tendencies toward dehumanization, are closely tied to factors related to work organization.

Our mediation analysis adds further empirical support for investigating the organizational level, while our results indicate that OD is associated with patient depersonalization both directly and indirectly via perceived stress (the indirect effect), our main finding lies in the retention of a highly significant direct effect between OD and patient depersonalization. Although this direct link has been demonstrated in other professional categories, such as prison officers [24], it had not yet been empirically tested for nursing staff.

Our findings resonate with the work of Baldissarri et al. [20], which established a link between employee objectification and burnout, wherein depersonalization serves as a defensive coping strategy. The persistence of a significant direct effect in our model suggests that perceived stress only partially accounts for the association between OD and patient depersonalization. Several theoretical mechanisms may explain this residual direct pathway, though our study design does not permit us to test them empirically.

First, drawing on prior work by Baldissarri et al. [20], self‐objectification wherein nurses internalize the organization’s instrumental view of their role may directly foster depersonalized attitudes toward patients independently of experienced stress. If nurses come to view themselves as tools or resources rather than as autonomous professionals, this mechanistic self‐perception may translate into a parallel perception of patients as objects of care rather than subjects with full humanity. However, we did not measure self‐objectification in the present study, so this remains a theoretical interpretation rather than an empirically supported pathway.

Second, the direct effect may reflect unmeasured mediators that operate in parallel to perceived stress. For instance, emotional exhaustion and cynicism, both core components of burnout not assessed here—are known to mediate relationships between organizational stressors and depersonalization [45]. OD may deplete nurses’ emotional resources or foster workplace cynicism through routes independent of general perceived stress, thereby contributing to depersonalized patient care.

Third, it is possible that OD and depersonalization share common antecedents that were not controlled for in our cross‐sectional model. For example, unit‐level organizational climate, leadership quality, or peer support cultures may simultaneously shape both nurses’ perceptions of being dehumanized and their tendency to adopt depersonalized coping strategies. In such a scenario, the observed direct association would partially reflect shared contextual variance rather than a genuine direct pathway from OD to depersonalization.

Future research should explicitly measure these candidate mechanisms using validated scales for self‐objectification, emotional exhaustion, cynicism, and organizational climate. Moreover, longitudinal or experimental designs are essential to distinguish genuine mediation pathways from spurious associations arising from unmeasured confounders or reverse causation (e.g., nurses who tend toward depersonalization may also perceive their organization as more dehumanizing).

## 6. Strengths and Limitations

The cross‐sectional design of this study is a primary limitation, as it precludes the establishment of causal relationships and prevents the capture of dynamic changes over time. To fully elucidate the underlying mechanisms and identify robust risk factors, longitudinal research is indispensable.

It is important to acknowledge that OD accounts for only 7% of the variance in perceived stress (*R* ^2^ = 0.07). While this effect is statistically significant (*p* < 0.001) and theoretically meaningful given the coherence with COR theory, it indicates that OD represents only one contributor among many organizational and individual factors influencing nurse stress. This modest explanatory power has direct implications for how our findings should be interpreted and applied.

First, OD should be understood as one structural risk factor within a multifactorial stress etiology, rather than as a dominant or primary determinant. Our model deliberately isolates the OD‐stress relationship to test a specific theoretical pathway, but in reality, nurses’ stress arises from a complex interplay of workload intensity, shift patterns, interpersonal conflicts, lack of autonomy, emotional demands, and individual differences in coping resources. The variance unaccounted for by OD (93%) likely reflects these unmeasured organizational and personal factors.

Second, given the modest explanatory power, we have avoided characterizing OD as a ‘major psychosocial risk factor’ and now describe it as one psychosocial risk factor among others. Stronger characterizations would overstate the magnitude of OD’s independent contribution to stress based on the present evidence. While OD may interact synergistically with other stressors or exert effects through pathways not captured in our mediational model, our data do not support claims that addressing OD alone would substantially reduce nurse stress levels.

Third, future research should adopt a more comprehensive approach by incorporating multiple organizational stressors simultaneously (e.g., workload metrics, schedule control, leadership support, and staffing adequacy) alongside individual difference variables (e.g., resilience, self‐efficacy, and social support). Such models would clarify the relative weight of OD within the broader stress architecture and establish whether OD exerts unique variance beyond more proximal work conditions. Additionally, hierarchical or multilevel modeling could partition variance attributable to unit‐level versus individual‐level factors, providing insight into whether OD operates primarily as an organizational climate variable.

Fourth, although we conceptually distinguish organizational dehumanization, burnout, and depersonalization, these constructs remain empirically correlated. This overlap may inflate some of the associations observed in self‐report data and limits our ability to isolate the unique contribution of OD from more general burnout‐related processes.

Fifth, we did not include key individual and contextual variables such as resilience, emotional intelligence, unit type, or workload variability, which are likely to act as confounders or moderators. As a result, our mediation model should be interpreted as a simplified pathway rather than a comprehensive explanation of depersonalization. Finally, our use of social‐media‐based snowball sampling entails substantial self‐selection and coverage biases: nurses who chose to participate may differ systematically from nonparticipants in stress levels and work environments, which restricts the generalizability of our findings and precludes any claim to representativeness of the French nursing workforce.

In sum, while our findings establish OD as a statistically reliable correlate of stress, the low *R* ^2^ limits strong causal or policy claims and underscores the need for multivariate longitudinal designs to establish OD’s true etiological significance.

We also acknowledge that the exclusive use of self‐report questionnaires raises the possibility of common method variance. Although our constructs are theoretically distinct and measured with established scales, shared method and response tendencies may have inflated some of the associations. Future research should combine multiple data sources (e.g., supervisor ratings, patient‐reported outcomes, and objective indicators) to better disentangle substantive relationships from method effects.

The use of social media and snowball sampling also implies potential self‐selection and coverage biases, so the present results should not be generalized to all French nurses without caution. Future investigations should address these issues by adopting more rigorous designs, such as cohort studies, and integrating objective data collection methods, like direct observation of workplace conditions.

The study’s completion rate of 58.31% (291/499) is high and aligns with recent European web‐based surveys (e.g., [51]). However, given the distribution via email and social media, the true denominator (total number of nurses invited) is uncertain, which introduces a potential self‐selection bias and limits the generalizability of the findings. While this high completion rate mitigates concerns about severe nonresponse bias, it remains possible that nurses experiencing the highest levels of distress opted out, potentially leading to conservative estimates of burnout prevalence. The use of social media may have further exacerbated this bias by favoring digitally engaged or younger staff, thus challenging the representativeness of the sample.

To enhance future studies, researchers should consider combining digital recruitment with face‐to‐face reminders or employing stratified sampling to improve representativeness. Furthermore, the exclusion of participants who did not complete all core modules may have introduced an additional layer of bias; ensuring a higher completion rate across all modules in subsequent research is advisable.

Finally, our model did not consider potential boundary conditions. It is likely that individual and contextual factors, such as nurses’ resilience, emotional intelligence, unit type, or workload variability, shape the strength of the relationships between OD, stress, and patient depersonalization. Future research should explicitly test these moderating effects using larger samples and longitudinal designs.

## 7. Recommendations for Future Research

The associations documented in this study point to several research priorities for further clarifying the relationship between OD and adverse patient‐directed outcomes.

The first priority is methodological. Because our cross‐sectional design cannot establish temporal precedence among OD, perceived stress, and depersonalization, longitudinal studies are needed to test the directional ordering implied by the spillover model. Multiwave designs would allow researchers to examine whether changes in perceived OD precede changes in stress and depersonalization, or whether reciprocal influences are at play. Reliance on self‐report instruments should also be tempered by the integration of multisource measures, including objective physiological markers of stress and organizational indicators such as turnover rates, absenteeism, and patient‐reported quality of care, in order to disentangle substantive associations from common method effects.

The second priority concerns the mechanisms of the partial mediation. Because perceived stress only partially accounts for the OD–depersonalization association, the residual direct relationship invites further empirical investigation. Future research should test more comprehensive mediation models that include the full spectrum of burnout components, particularly emotional exhaustion and cynicism, as well as candidate constructs such as self‐objectification, psychological flexibility, and perceived meaningfulness in work. Whether these constructs operate as additional mediators or as moderators that buffer the impact of OD remains an open empirical question.

Finally, the external validity of the present findings would be strengthened by recruitment strategies that go beyond social‐media‐based snowball sampling. Stratified sampling across facility types, regions, and care contexts within the French healthcare system, as well as comparative analyses with healthcare systems operating under different funding and governance models, would help clarify whether the patterns observed here generalize across organizational arrangements.

## 8. Policy and Practice Implications

The associations documented in this study suggest several directions for health policy, hospital governance, and clinical practice, although the cross‐sectional and nonprobability nature of our design precludes firm policy prescriptions on the basis of these data alone. The implications outlined below should therefore be read as hypothesis‐generating rather than as definitive recommendations and would need to be substantiated by longitudinal and multisource evidence before being translated into formal policy.

If the associations reported here reflect underlying causal relationships, OD may warrant consideration as one psychosocial risk factor among others contributing to nurse stress and to the relational quality of care. Recognizing OD alongside more proximal factors such as workload, staffing levels, and shift organization would shift part of the analytic frame from individual coping toward organizational structure, without implying that OD is a dominant or sufficient cause of either nurse stress or patient depersonalization.

At the level of clinical and managerial practice, the present findings are consistent with the broader literature suggesting that interventions targeting organizational sources of dehumanization may complement, rather than replace, interventions aimed at individual stress management. Hospital administrators and nurse managers could review performance indicators and staffing policies that convey a purely instrumental view of nurses, for instance, an exclusive emphasis on productivity or time‐based indicators, and consider restoring protected time for relational care, clinical judgment and ethical reflection.

In daily practice, candidate organizational changes that align with this perspective include limiting excessive task fragmentation, adjusting nurse‐to‐patient ratios where staffing constraints allow, and creating regular spaces for team debriefing in which nurses can discuss ethically challenging situations without fear of sanction. Whether such changes would translate into measurable reductions in perceived dehumanization, stress, and depersonalization is itself an empirical question that intervention studies are best positioned to address.

## 9. Conclusion

This study examined the cross‐sectional associations linking OD, perceived stress and patient‐directed depersonalization in a sample of 291 French nurses, and tested whether perceived stress statistically mediates the OD–depersonalization association as proposed by the spillover model.

Consistent with the theoretical framework, OD was significantly associated with perceived stress, and perceived stress partially mediated the association between OD and depersonalization. The OD–stress association was robust to common method variance correction, while the stress–depersonalization association showed modest attenuation. The size of these associations, however, is modest: OD accounted for approximately 7% of the variance in perceived stress and, together with stress, for approximately 7.5% of the variance in depersonalization. The cross‐sectional design and nonprobability sample further limit the inferences that can be drawn from these associations.

Rather than establishing a causal pathway from organizational structure to patient outcomes, our results provide preliminary empirical support for one component of a broader theoretical model in which institutional treatment of staff is hypothesized to shape, in part, the relational quality of care. The ethical observation that the dignity afforded to caregivers and the dignity afforded to patients are normatively connected does not depend on the magnitude of the statistical effects reported here, but the empirical claim that addressing OD is a productive lever for reducing patient depersonalization requires longitudinal and intervention‐based evidence that this study cannot provide. We therefore frame our findings as a basis for further investigation rather than as a foundation for definitive policy or practice claims.

## Author Contributions

Marie Charlotte Mollet: conceptualization, data collection, formal analysis, and writing–original draft; Oulmann Zerhouni, Lucia Romo, and Corinne Isnard Bagnis: validation and writing–review and editing.

## Funding

No funding was received for this manuscript.

## Disclosure

The researchers maintained full autonomy over all stages of the study, including the experimental design, data acquisition, statistical analysis, interpretation of findings, and the final decision to submit the work for publication.

## Ethics Statement

This study was conducted in strict accordance with the ethical standards of the relevant national and institutional research committees, as well as the 1964 Declaration of Helsinki and its subsequent amendments. As a psychological research project, this study adhered to all international ethical guidelines regarding human participants and minimal‐risk protocols.

## Consent

Prior to their participation, informed consent was secured from every individual involved in the study.

## Conflicts of Interest

The authors declare no conflicts of interest.

## Supporting Information

Additional supporting information can be found online in the Supporting Information section.

## Supporting information


**Supporting Information** Appendix 1: STROBE Statement—Checklist of items that should be included in reports of cross‐sectional studies.

## Data Availability

The data that support the findings of this study are available from the corresponding author upon reasonable request.
