# Supplementary material for: Organizational Dehumanization, Perceived Stress, and Patient Depersonalization in French Nurses: A Cross‐Sectional Test of the Spillover Model
Source: J Nurs Manag. 2026 Jun 22;2026:2207579. doi: 10.1155/jonm/2207579 (PMC13287826; doi:10.1155/jonm/2207579)
Supplement: Supplementary file 1 — Supporting Information Appendix 1: STROBE Statement—Checklist of items that should be included in reports of cross‐sectional studies. [file JONM-2026-2207579-s001.docx]

Appendix 1 : The STROBE checklist

**Item 1, Title and abstract – Study design stated in title and in abstract first sentence; informative, balanced summary provided.**

**Item 2, Background/rationale – Scientific background and rationale explained .**

**Item 3, Objectives – Aim and four objectives stated explicitly in the Abstract and Introduction.**

**Item 4, Study design – Key elements of design presented early in Methods.**

**Item 5, Setting – Description of setting, locations and relevant dates, including period of data collection .**

**Item 6, Participants – Eligibility criteria and recruitment procedures detailed.**

**Item 7, Variables – All outcomes, exposures, predictors, potential confounders and effect modifiers defined, including diagnostic criteria where applicable.**

**Item 8, Data sources/measurement – Sources of data and methods of assessment for each variable described, with reliability/validity evidence.**

**Item 9, Bias – Efforts to address potential sources of bias discussed.**

**Item 10, Study size – Sample-size considerations explained.**

**Item 11, Quantitative variables – Handling of quantitative variables and any groupings described.**

**Item 12, Statistical methods – All statistical methods, including cluster derivation, validation checks, handling of missing data and sensitivity analyses, specified.**

**Item 13, Participants – Numbers at each stage of study and flow diagram (Appendix Figure A1) noted.**

**Item 14, Descriptive data – Characteristics of study participants and information on exposures and potential confounders provided.**

**Item 15, Outcome data – Numbers of outcome events or summary measures reported for each profile.**

**Item 16, Main results – Unadjusted and adjusted estimates with precision and reference category stated; p-values and effect sizes provided.**

**Item 17, Other analyses – Additional analyses (e.g., sensitivity checks, subgroup comparisons) described in the results section.**

**Item 18, Key results – Summary of key results with reference to objectives**

**Item 19, Limitations – Study limitations, potential bias and imprecision discussed**

**Item 20, Interpretation – Overall interpretation of results in context of objectives and relevant evidence**

**Item 21, Generalisability – External validity and applicability of findings considered**

**Item 22, Funding – Source of funding and role of funders stated**
